# Assessment of Long-Term Engagement in Aerobic Versus Resistance Exercise on 20-Year Cardiovascular Disease Incidence (2002–2024): The ATTICA Epidemiological Cohort Study

**DOI:** 10.3390/jcm14020522

**Published:** 2025-01-15

**Authors:** Nikos Dimitriadis, Giannis Arnaoutis, Christina Chrysohoou, Fotios Barkas, Evangelos Liberopoulos, Petros P. Sfikakis, Christos Pitsavos, Costas Tsioufis, Konstantinos D. Tambalis, Demosthenes Panagiotakos

**Affiliations:** 1 Department of Nutrition and Dietetics, School of Health Sciences and Education, Harokopio University, 176 76 Athens, Greece; nikidimi@hua.gr (N.D.); garn@hua.gr (G.A.); dbpanag@hua.gr (D.P.); 2 First Cardiology Clinic, School of Medicine, National and Kapodistrian University of Athens, Hippokration Hospital, 115 28 Athens, Greece; chrysohoou@usa.net (C.C.); cpitsavo@med.uoa.gr (C.P.); kptsioufis@gmail.com (C.T.); 3 Department of Internal Medicine, Medical School, University of Ioannina, 451 10 Ioannina, Greece; f.barkas@uoi.gr; 4 First Department of Propaedeutic Internal Medicine, Medical School, National and Kapodistrian University of Athens, Laiko General Hospital, 157 72 Athens, Greece; elibero@med.uoa.gr (E.L.); psfikakis@med.uoa.gr (P.P.S.); 5 School of Physical Education and Sports Science, National and Kapodistrian University of Athens, 115 28 Athens, Greece

**Keywords:** physical activity, aerobic, resistance, cardiovascular disease, risk

## Abstract

**Objective**: The aim of this study was to assess whether aerobic exercise, as opposed to resistance training or a combination of both, is associated with long-term cardiovascular outcomes. **Methods**: The ATTICA study is a population-based cohort study with a 20-year follow-up (2002–2022); it was conducted in the Attica region, Greece, and included 3042 adult participants (45 ± 11 years, 1518 females). Physical activity engagement in aerobic, resistance, or combined exercise, and 20-year tracking, together with information regarding atherosclerotic cardiovascular disease (ASCVD) incidence were available for 1988 participants (45 ± 12 years old, 987 males, 1001 females). Physical activity levels were evaluated using the validated International Physical Activity Questionnaire, in all follow-up examinations (2001–2002, 2006, 2012, and 2022). Cox proportional hazard models were applied; the results are presented as hazard ratio (HR) and 95% confidence intervals (CIs). **Results**: Participants engaged in the combined (aerobic and resistance) physical activity group had 0.41-times [95%CI (0.20, 0.82)] lower ASCVD risk compared to inactive participants; participants in the healthy engaging aerobic physical activity (HEPA) group had 0.54-times [95%CI (0.36, 0.80)] lower ASCVD risk; no significant associations were observed regarding the minimally active aerobic group [HR, 0.81, 95%CI (0.57, 1.17)], or the resistance training only group [HR, 1.17, 95%CI (0.25, 1.52)]. **Conclusions**: These findings carry a strong public health message, underscoring the importance of incorporating aerobic or combined aerobic–resistance training into physical activity guidelines to enhance cardiovascular health and reduce the long-term risk of CVD.

## 1. Introduction

Regular physical activity is essential for maintaining overall health, reducing the risk of chronic diseases, and enhancing physical and mental well-being across all age groups. The contribution of low physical activity to atherosclerotic cardiovascular disease (ASCVD) and all-cause mortality, measured in DALYs, has increased by 83.9% and 82.9%, respectively, since the 1990s [1,2,3]. The guidelines from the European Society of Cardiology (ESC) strongly recommend (i.e., Class I, Level A) that adults of all ages should strive for at least 150–300 min a week of moderate-intensity or 75–150 min a week of vigorous-intensity aerobic physical activity, or an equivalent combination thereof [1], which is in line with the American College of Cardiology/American Heart Association Task Force on Clinical Practice Guidelines [2].

While the health benefits of aerobic physical activity are well-documented, the impact of resistance training on atherosclerotic cardiovascular disease (ASCVD) risk remains less well understood and appreciated. The European Society of Cardiology (ESC) recommends engaging in resistance exercises at least twice per week, targeting all major muscle groups with 8–10 different exercises. Each exercise should comprise 1–3 sets of 8–12 repetitions, performed at an intensity of 60–80% of the individual’s one-repetition maximum. This regimen has been shown to significantly reduce blood pressure but has not been conclusively linked to a reduction in ASCVD risk [2]. Evidence from basic and clinical research indicates that resistance training improves muscular fitness, endothelial function, triglyceride metabolism, and insulin sensitivity [4,5,6]. However, data on its effects on other cardiovascular risk factors and ASCVD risk remain limited. As our understanding of various physical activity modalities evolves, it is plausible that their effects on the cardiovascular system could complement one another, potentially influencing individualized exercise recommendations. For now, however, the role of regular resistance exercise in ASCVD prevention continues to be an area of active investigation [6,7].

To the best of our knowledge there is no study evaluating the long-term impacts of aerobic versus resistance, or in combination with aerobic, physical activity on cardiovascular risk in people from the general population. Thus, the aim of this study was to assess whether aerobic exercise, as opposed to resistance training or a combination of both, is associated with long-term ASCVD outcomes. To test this hypothesis, we analyzed physical activity trajectory data from the 20-year (2002–2022) follow-up of the ATTICA study [8,9]. Specifically, we hypothesized that individuals who engaged in a combination of aerobic and resistance exercises would experience the greatest benefits from maintaining an active lifestyle, particularly in reducing CVD mortality and morbidity.

## 2. Methods

Information about the objectives, design, sampling procedure, and methodology of the ATTICA study have been presented in previously published papers [8,9,10].

### 2.1. Study Design

The ATTICA study is a prospective cohort (cohort study). It has been designed to assess the distribution and long-term trends in various socio-demographic, lifestyle, clinical, biochemical, and psychological factors associated with CVD, as well as the relationships between these factors and CVD incidence.

### 2.2. Setting

The study was conducted in the Attica region of Greece; the area (with a total population of 3,5 million inhabitants) encompasses 58 municipalities, including the capital city, Athens. This region comprises 78% urban areas, with the remainder classified as rural. In 2001, a total of 4056 individuals were randomly selected from municipality records and employment listings and invited to participate in the study. The sampling process was stratified according to the sex and age group distribution of the region, as outlined in the 2001 census. Preliminary evaluations for exclusion criteria (detailed below) were conducted at participants’ residences or workplaces by the study’s physicians and healthcare professionals.

### 2.3. Sample

Out of the 4056 who were initially invited, 3042 individuals (1514 males, mean age 46(13), 1518 females, mean age 45(14)) who were free of CVD, cancer, or chronic inflammatory diseases and were not living in institutions (i.e., exclusion criteria as described in the baseline paper, [8]) and voluntarily agreed to participate comprised the study’s sample (75% participation rate).

### 2.4. Follow-Up

Three follow-up assessments were conducted at 5 years (2006), 10 years (2012), and 20 years (2022) after the baseline evaluation (2001–2002). At each follow-up, the occurrence of ASCVD or other conditions (including fatal events) was recorded, along with clinical status, lifestyle factors (e.g., dietary habits, physical activity, and smoking), and psychological well-being, using consistent methodologies [9,10]. Specifically, 2583 participants were located and consented to re-examination at the 10-year follow-up in 2012 (85% participation), while 2169 participants were re-examined at the 20-year follow-up in 2022 (71% participation). Among the 873 individuals lost to follow-up, 771 could not be contacted due to changes in contact information or errors in addresses or phone numbers, and 102 declined to participate. For deceased participants, information was obtained from relatives and death certificates. A comparison of the age and sex distribution of the follow-up samples with the baseline group showed no significant differences (*p*-values > 0.80).

### 2.5. Bioethics

The study adheres to the ethical principles established in the Declaration of Helsinki. The Ethics Committee of the First Cardiology Department of the National and Kapodistrian University of Athens (#017/01.05.2001) and the Ethics Committee of Harokopio University (#38/29.03.2022) approved the study. All participants were informed about the objectives and procedures, providing their consent prior to participation.

### 2.6. Measurements

#### 2.6.1. Physical Activity Status Assessment

A version of the International Physical Activity Questionnaire—Short Form (IPAQ-SF) translated into Greek was utilized to assess participants’ weekly energy expenditure and determine their engagement in aerobic training [11,12]. The frequency, duration, and intensity of leisure-time aerobic exercise during a typical week were recorded. Intensity was quantified using Metabolic Equivalent (MET) values, where 1 MET represents the energy expenditure at rest (approximately 3.5 mL of oxygen per kg of body weight per minute [mL/kg/min]). Participants were then classified into three groups, (a) inactive, (b) minimally active, and (c) engaging in health-enhancing physical activity (HEPA), based on the frequency and duration of moderate and vigorous activities, following the IPAQ scoring guidelines [11]. Furthermore, based on a methodology described in a previous publication of the study by Tambalis et al. [13] participants were classified (only or in combination with aerobic) on resistance exercise (isometric, isotonic, or isokinetic); the frequency and duration of resistance training was recorded. Thus, 3 groups of physical activity were defined in the study’s sample: (i) minimally or HEPA aerobic only, (ii) resistance only, and (iii) combined aerobic and resistance. Moreover, using the information retrieved through the follow-up examinations, 4 trajectories of physical activity were defined based on the tracking over 2002–2022 of physical activity status. Participants were classified as (a) consistently inactive if they reported sedentary lifestyle or engagement in light physical activity in all three follow-up examinations held in 2006, 2012, and 2022; (b) became inactive from physically active if reported sedentary lifestyle or engagement in light physical activity in the follow-up examinations held in 2012 or 2022; (c) became active from physically inactive if they reported engagement in physical activities in all follow-up examinations held in 2012 or 2022; or (d) consistently active during the entire period 2002–2022 [14].

#### 2.6.2. Socio-Demographic, Lifestyle, Biochemical, and Clinical Characteristics Assessment

Several other characteristics of the participants were also recorded at baseline examination, including place of residence, highest educational level achieved, smoking status (current/ever, and pack-years of smoking, i.e., average cigarettes per day per year), dietary habits (measured through a validated semi-quantitative food frequency questionnaire [15]), clinical assessment regarding resting blood pressure, and anthropometric indices and medical history, as well as blood lipids, triglycerides, and inflammatory marker levels. Participants were classified as follows: those with total serum cholesterol levels exceeding 200 mg/dL or on lipid-lowering medication were identified as having hypercholesterolemia; those with blood sugar levels above 125 mg/dL or using antidiabetic medication were classified as having diabetes mellitus; and those with average systolic/diastolic blood pressure ≥140/90 mmHg or on antihypertensive medication were categorized as having hypertension. Body mass index (BMI) was calculated based on measured weight and height, with obesity defined as a BMI greater than 29.9 kg/m^2^.

Adherence to a Mediterranean-type diet was evaluated using the MedDietScore (ranges from 0 to 55 points; the higher the values, the greater the adherence to the traditional dietary pattern) [16].

#### 2.6.3. Follow-Up Assessment

The development of fatal or non-fatal ischemic heart disease, stroke, or any other type of ASCVD was evaluated during all follow-up examinations by the study physicians, based on the International Classification of Diseases (ICD)-10 guidelines. For individuals who experienced multiple cardiovascular events (e.g., an initial stroke or heart failure followed by coronary heart disease), the first event was recorded as the primary endpoint. Subsequent events were used for additional analyses to assess potential competing risks.

### 2.7. Statistical Analysis

Continuous variables are presented as means with standard deviations, while categorical variables are presented as relative frequencies (percentages). The associations between categorical variables were examined using Pearson’s chi-squared test. To compare the mean values of continuous variables, Analysis of Variance with the F-test was used, or the Kruskal–Wallis test for non-normally distributed data was used, with Levene’s test assessing the equality of variances. When variances were unequal, Welch’s F-test was applied. Adjustments for multiple between-group comparisons were made using the *t*-test or U-test, with Bonferroni correction used to control for *p*-value inflation. The normality of variables was assessed using P-P plots. The cumulative incidence of ASCVD was calculated as the ratio of new cases to the total number of available cases in each group. The *p*-values for comparisons between physical activity groups or trajectories were determined using the Z-test for equality of proportions with continuity correction. The direct effect of physical activity trajectories on ASCVD incidence was evaluated using Cox proportional hazards models, with proportionality assumptions tested graphically. The results are presented as hazard ratios (HRs) and their 95% confidence intervals (CIs); all models were adjusted for age, sex, and baseline CVD risk factors. Goodness-of-fit statistics of the estimated models included likelihood ratio (LR), which was evaluated through chi-squared test, and the Akaike Information Criterion (AIC). Higher LR values indicate a better fit, while lower AIC values indicate a better model. Nagelkerke’s R^2^, a measure analogous to R^2^ in linear regression modeling, allows values ranging between 0 and 1 and shows to what extent the factors included in a model may predict the outcome. The log-rank test was used to compare cumulative incidence between groups. The Sobel–Goodman test was applied to evaluate any potential mediating effect in the relationship between physical activity level and CVD incidence, and to calculate what percent of the effect of physical activity on CVD risk is explained by the indirect effect of physical activity on BMI, lipid and inflammation markers, and the clinical CVD risk factors, i.e., hypertension, diabetes, and hypercholesterolemia [17]. *p*-values were based on two-sided hypotheses. Statistical analyses were conducted using STATA 17 (STATA Corp, College Station, TX, USA).

## 3. Results

### 3.1. Participants’ Characteristics According to Trajectories of Physical Activity Status

At baseline examination, 39% of males and 33% of females were defined as physically active, as well 31% males and 26% females at 5-year follow-up (in 2005), 26% males and 19% females at 10-year follow-up (in 2012), and 30% males and 32% females at 20-year follow-up (in 2022). Moreover, 47% of the participants were classified as always inactive during the 2002–2022 period, 23% became inactive from physically active, 18% became active and, only 9% of males and 15% of females sustained physical activity levels (*p* < 0.001). In a previous publication of the ATTICA study, participants’ baseline characteristics according to the 20-year trajectories of physical activity status were presented in detail [14].

### 3.2. Participants’ Characteristics According to Physical Activity Groups

In Table 1, participants’ characteristics according to physical activity groups, i.e., aerobic only, resistance training only, and combined, are presented. Participants that were engaged in resistance training (alone or in combination with aerobic) were younger than the rest, and they had lower reports of smoking habits, and a better lipid and triglycerides profile; all participants involved in any type, aerobic or resistance exercise, at any level had lower BMI levels as compared to the physically inactive group. Only participants in the aerobic HEPA and combined exercise had lower hs-CRP levels. These associations were considered to mediate potential residual confounding in the survival analyses.

### 3.3. Assessment of 20-Year Incidenceof Cardiovascular Disease in Relation to Aerobic, Resistance, or Combined Exercise

Of those participants who were defined as physically active, consistently or at any time point [at any point] during the 20-year follow-up, 1271 (42%) were engaged only in aerobic training (minimal or HEPA), 37 (1.2%) only in resistance training, and 168 (5.5%) in combined aerobic and resistance training. No significant differences were observed between genders regarding the distribution of types of physical activity (*p* = 0.657). The 20-year cumulative incidence of CVD was 375 cases/940 participants (39.9%) who were consistently physically inactive versus 194 cases/497 participants (39.0%) who were engaged at minimal aerobic exercise group (age, sex adjusted *p* < 0.001), 118 cases/390 participants (30.3%) who were engaged at HEPA aerobic exercise group (*p* < 0.001), 7 cases/30 participants (23.3%) who were engaged at resistance training group (*p* = 0.067), and 24 cases/131 participants (18.3%) who were engaged at combined aerobic and resistance exercise group (*p* < 0.001). No gender differences in terms of CVD incidence could be tested between physical activity groups due to the small number of cases in each group.

Age- and sex-adjusted survival analysis revealed that participants in the combined aerobic and resistance exercise group had 0.41-times [95%CI (0.20, 0.82)] lower risk of developing ASCVD during the 20-year of follow-up, as compared to the inactive group. Moreover, participants in the HEPA aerobic exercise group had 0.54-times [95%CI (0.36, 0.80)] lower risk of developing CVD as compared to the inactive group. No significant associations were observed regarding the minimally active aerobic group [HR, 0.81, 95%CI (0.57, 1.17)] or the resistance training only group [HR, 1.17, 95%CI (0.25, 1.52)], as compared to the inactive group.

**Figure 1:**
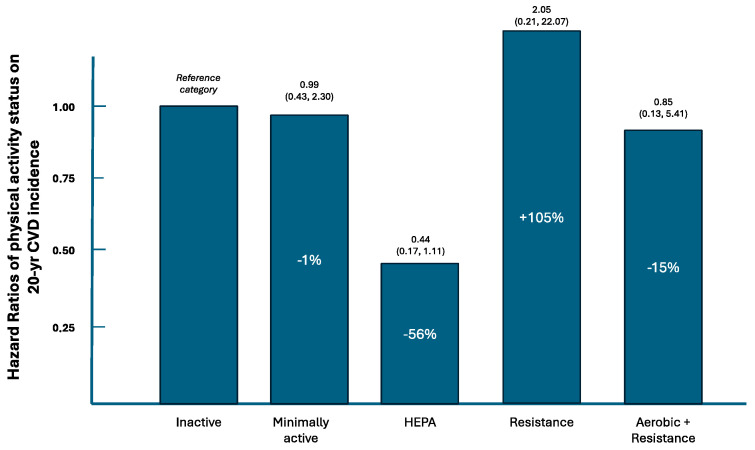
Hazard ratios and attributable risk (as %) of 20-year CVD incidence due to physical activity among healthy participants of the ATTICA study (2002–2022).

However, residual confounding may still exist as several associations between physical activity groups and several baseline participants characteristics (Table 1). Thus, nested survival models were estimated; the basic *Model 1* was adjusted for age, sex, and MET-minute/week, to account for the age differences observed between groups and energy expenditure. It was revealed that the HEPA group and the combined aerobic and resistance training group had a lower risk of developing CVD, as compared to the physically inactive group. Then, in *Model 2,* social and lifestyle factors were considered, without altering the previous findings, suggesting a beneficial association of HEPA, as well as the combined aerobic and resistance training, towards CVD risk. However, when intermediate biochemical CVD risk markers were entered in *Model 3*, physical activity was not associated with CVD risk. Similarly, all significant associations were lost when medical history and management (i.e., treated or untreated) of hypertension, diabetes, and hypercholesterolemia were also considered (*Model 4*) (Table 2). In addition, when aerobic HEPA or combined aerobic and resistance physical activity was compared to the resistance training group, the risk for CVD was reduced by 22% [HR 0.78, 95%CI (0.53, 1.01)] and by 29.7% [HR 0.70, 95% CI (0.37, 1.02)], respectively. The hazard ratios and attributable risks are illustrated in Figure 1.

### 3.4. Mediation Analysis

The later findings underline the need for further mediation analysis to explore the mechanism by which HEPA or combined physical activity reduce CVD risk. The Sobel–Goodman test revealed that the association between aerobic and/or resistance exercise and CVD risk is mediated 21.7% through the indirect effect of physical activity on hypertension (*p* < 0.001), 15.5% through the indirect effect of physical activity on diabetes (*p* < 0.001), 22.1% through the indirect effect of physical activity on cholesterol levels (*p* < 0.001), and 5.5% through the indirect effect of physical activity on hs-CRP levels (*p* = 0.023).

## 4. Discussion

This study investigated whether aerobic exercise, resistance training, or a combination of both is associated with long-term cardiovascular outcomes. It was revealed that aerobic HEPA or combined aerobic and resistance training is associated with a substantial reduction in CVD incidence over the 20-year follow-up, with a superior effect on CVD risk reduction compared to resistance training alone. Despite the limitations carried on due to the observational design of the study, these findings carry a strong public health message, underscoring the importance of incorporating aerobic or combined aerobic–resistance training into physical activity guidelines to enhance cardiovascular health and reduce the long-term risk of CVD.

### 4.1. Relevant Studies Evaluating Aerobic, Resistance, or Combined Exercise in Relation to CVD Outcomes

The effects of aerobic exercise on cardiovascular physiology have been well studied over the past decades, revealing various pleiotropic benefits, such as regulating arterial blood pressure, lowering lipid and triglyceride levels, reducing inflammatory markers, and improving endothelial function [2]. However, relatively few studies have demonstrated the benefits of resistance exercise in reducing surrogate risk markers for atherosclerotic cardiovascular disease (ASCVD), as well as cardiovascular morbidity and mortality. A systematic review and meta-analysis on the lipid profiles of healthy women found that combined exercise training improved triglyceride and total cholesterol levels, contributing to optimal cardiovascular health [18,19]. Similarly, a network meta-analysis by Liang et al. showed that combined exercise was most effective in controlling blood glucose and triglyceride levels, while resistance exercise was most efficient in improving LDL cholesterol levels [20]. Consistent with these findings, Lee et al. reported that aerobic exercise, either alone or combined with resistance training, improved the composite cardiovascular risk profile compared to the control group, which included either resistance exercise alone or no physical activity [21]. Our findings on the associations between various types of physical activity and clinical and biochemical markers showed that the HEPA group and the combined physical activity group exhibited lower levels of HDL-C and hs-CRP compared to the resistance exercise and combined training groups. Additionally, engaging in HEPA or combined aerobic and resistance training was linked to a significant reduction in CVD incidence over a 20-year follow-up period, demonstrating a more pronounced effect on reducing CVD risk than resistance training alone. It was also found that resistance training improves triglycerides, total cholesterol, and LDL-cholesterol (see Table 1), but these changes were insufficient to reduce the risk of ASCVD (see Table 2).

### 4.2. Recommendations Regarding Physical Activity and CVD

The European Society of Cardiology advise that “*all adults should be engaged in regular aerobic physical activities to reduce all-cause mortality, as well as ASCVD-specific mortality, and morbidity*” [1], which is in line with the World Health Organization 2020 guidelines on physical activity and sedentary behavior [22]. Certain individuals that are unable to reach the minimum levels of physical activity due to various disadvantages in health and abilities should strive to minimize sedentary behavior throughout their day and be as active as they are able to be [1]. For older adults or individuals with chronic conditions who are unable to meet the recommended 150 min of moderate-intensity physical activity per week, engaging in physical activity to the extent permitted by their abilities and health status is encouraged. The 2019 joint guidelines from the American College of Cardiology and the American Heart Association also strongly advocate for routine counseling during healthcare visits to promote an active lifestyle among all adults. Additionally, reducing sedentary behavior is considered a reasonable strategy for lowering the risk of atherosclerotic cardiovascular disease (ASCVD), (class IIb, level C) [3]. Consistent with previous guidelines, the American College of Sports Medicine (ACSM) recommends that adults participate in moderate-intensity aerobic exercise and perform resistance training approximately 2–3 times per week [23].

### 4.3. Pathophysiological Mechanisms Regarding Aerobic and Resistance Exercise and CVD

While aerobic exercise is widely recognized as a primary cardioprotective lifestyle intervention for reducing ASCVD risk, some studies suggest that adding resistance exercise to aerobic activity may provide additional benefits for ASCVD risk reduction. However, the independent effects of resistance exercise alone on ASCVD risk remain unclear and underappreciated [2,6,7]. Resistance training primarily improves muscular strength and bone density. Evidence from interventional and experimental studies indicates that resistance exercises, including weightlifting and bodyweight exercises, enhance energy expenditure, enhance muscle mass, and elevate metabolic rate, thereby supporting weight management and reducing the risk of obesity [24]. Resistance exercise has also been associated with better blood pressure regulation [25], a finding that was observed in our analyses, too, both at baseline and during the 10-year follow-up examination. Moreover, several clinical trials have shown that engagement in resistance exercise has improved individuals’ lipid profile by increasing levels of HDL-cholesterol and reducing levels of LDL-cholesterol and triglycerides, which was also evident in the present study as well. Resistance training has also been shown to enhance insulin sensitivity, enabling cells to respond more effectively to insulin and regulate blood sugar levels. Additionally, it reduces levels of inflammatory markers, which are closely linked to the development and progression of ASCVD [26]. Our study also revealed that participants engaged in resistance or combined exercise training had a significantly lower prevalence and 10-year incidence of diabetes. A few studies have also demonstrated that resistance exercise can enhance endothelial function [27]. It is important to highlight that the pressure load exerted on the heart during resistance exercise can lead to a mild form of cardiac hypertrophy. Additionally, resistance exercise can cause a substantial increase in blood pressure, which may have adverse effects on individuals with uncontrolled hypertension, particularly when performing high-intensity resistance activities [27]. It should be underlined here that all significant associations between physical activity status and 20-year CVD incidence were lost when medical history and management of hypertension, diabetes, and hypercholesterolemia were considered in the analyses. This finding suggests that these medical conditions and their management may play a more dominant role in determining long-term CVD risk, potentially attenuating the independent protective effect of physical activity. As discussed earlier, physical activity influences CVD risk partly by reducing hypertension, improving glycemic control, and lowering cholesterol levels. However, if these factors are already well managed (e.g., through medication or lifestyle interventions), the independent contribution of physical activity to CVD risk reduction may be diminished. Specifically, the association between physical activity and reduced CVD risk might be partly explained by its effect on these conditions. Adjusting for their management isolates their impact, revealing that the observed benefits of physical activity could largely be mediated through improvements in these risk factors.

### 4.4. Limitations

This study offers several strengths; however, it also has notable limitations that should be taken into account when interpreting the results. The observational design of the study carries on residual confounding that may persist despite the adjustments made for known factors, as unmeasured variables or imperfectly measured covariates could still affect the observed associations. The lack of stratified analyses on the specific types of CVD outcomes, such as ischemic heart disease, stroke, or heart failure, may compromise the generalization of the results. The fact that there are no comparative analyses across these groups for developed pathologies may raise a question in view of whether the findings have to do primarily with the improvements in dyslipidemia and hypertension or represent an extension to broader cardiovascular outcomes. Moreover, as the mortality rate was relatively low in this cohort, we did not have adequate statistical power to perform comparisons between the physical activity groups in relation to CVD mortality. Furthermore, the resistance-only group comprised only 1.2% of participants, limiting robust statistical comparisons for the generalization of results. One other limitation is the reliance on self-reported physical activity data collected through the International Physical Activity Questionnaire. While widely used, this method is subject to recall bias, as participants may inaccurately remember or overestimate their activity levels. Additionally, self-reports are prone to social desirability bias, where individuals might exaggerate their physical activity to align with perceived societal norms or expectations. These inaccuracies can lead to misclassification of activity levels, potentially affecting the validity of the findings and weakening the observed associations between physical activity and CVD incidence. However, it should be noted that this tool has been shown to be reliable, repeatable, and widely used in epidemiological research. Another limitation of the study is the lack of precise, objective measurements to assess strength during resistance exercises. The reliance on indirect or self-reported data for such activities can lead to inaccuracies, as it does not capture the intensity, frequency, or progression of resistance training. Objective methods, such as one-repetition maximum testing or standardized strength assessments, would have provided more reliable and detailed information about participants’ muscular strength and effort during resistance exercises. This absence of precise evaluation limits the ability to fully understand the relationship between resistance training and CVD incidence or other health outcomes. Future studies could use actigraph accelerometers and similar devices to provide more accurate and objective data on physical activity levels compared to self-reported measures. Regarding other potential sources of risk of bias, factors such as selection bias, misclassification of participants to lifestyle categories, and residual confounding due to unmeasured factors should also be considered. Finally, although the loss to follow-up rate was within the acceptable levels for observational studies, it may have introduced some bias into the data analysis.

## 5. Conclusions

By highlighting the greater efficacy of aerobic and combined training in CVD prevention, this study supports a strategic approach to exercise interventions, which can play a pivotal role in public health efforts aimed at reducing the burden of CVD across diverse populations.

## Figures and Tables

**Table 1:** Participants’ characteristics at baseline and 10-year follow-up examination, according to physical activity groups, aerobic only, resistance training only, and combined (*n* = 1988).

	Inactive	Aerobic Activities		Resistance Training	Aerobic and Resistance Training	*p*
		Minimally Active	HEPA			
Νo. participants	940	497	390	30	131	
Males, *n*	463	258	68	15	60	
Age (years)	46 (13)	45 (15)	49 (15)	38 (12) *	39 (13) *	<0.001
Education, years of school	12(4)	13(4) *	12(4)	12 (3)	13(3)^*^	0.0165
Ever smoking, %	59	53	50	46	48	0.003
Pack-year of smoking	28(25)	21 (25)	28 (35)	17 (20) *	15 (15) *	<0.001
MET-minute/week	163 (88)	1518 (663) *	3614 (1318) *	1843 (2210) *	3006 (2384) *	<0.001
MedDietScore (0–55)	25 (7)	26 (7)	25 (5)	25 (5)	27 (6)	0.084
Body mass index (kg/m^2^)	27.8(4.6)	25.9(4.3) *	25.8 (4.1) *	25.9 (3.7) *	24.3 (3.3) *	<0.001
Hypertension, %	32	30	28	13 *	18 *	0.0006
Hypertension at 10 yr, %	59	53	52	37 *	35 *	<0.001
Diabetes, %	8.5	5.1	7.1	2.7 *	0.5 *	<0.001
Diabetes at 10 yr, %	27	19	21	17 *	5 *	<0.001
Hypercholesterolemia, %	40	40	40	29 *	28 *	0.0287
Hypercholesterolemia at 10 yr, %	67	65	63	54 *	53 *	0.0251
Triglycerides (mg/dL)	121(88)	114 (74)	136 (77)	100 (55) *	91 (56) *	0.001
Total cholesterol (mg/dL)	195(42)	193 (41)	194(38)	177 (37) *	180 (40) *	0.0001
HDL-cholesterol (mg/dL)	44.7 (15.1)	48.9 (13.4)	50.1 (13.0) *	47.9 (11.4)	51.0 (15.0) *	0.032
LDL-cholesterol (mg/dL)	123 (37)	123 (37)	121 (34)	112 (37) *	108 (35) *	0.019
hs-CRP (mg/L)	2.1 (2.5)	1.8 (2.3)	1.4 (1.7) *	1.9 (3.0)	1.6 (2.3) *	0.0008
IL-6 (pg/mL)	1.5 (0.5)	1.4 (0.4)	1.5 (1.1)	1.5 (0.9)	1.4 (0.8)	0.276

Data are expressed as mean (standard deviation) or frequencies (%). Overall *p*-values derived using ANOVA, Kruskal–Wallis, or chi-squared tests; post hoc analyses vs. the physically inactive group (reference category) were performed using the *t*-test or the chi-squared test, and the corresponding *p*-values (* denotes *p*-value < 0.05) were corrected using the Bonferroni rule. Abbreviations: HEPA, health enhancing physical activity; LDL-C: low-density lipoprotein cholesterol (calculated via Friedewald equation); HDL-C: high-density lipoprotein cholesterol; hs-CRP: high sensitivity C-reactive protein; IL-6: interleukin-6.

**Table 2:** Results (HRs and 95%CIs) from age- and sex-adjusted nested survival models that evaluated the association between types of physical activities in relation to the development of CVD during a 20-year period (2002–2022) in the ATTICA study participants.

	*Model 1*	*Model 2*	*Model 3*	*Model 4*
Physical activity groups				
*Minimally* active vs. inactive	0.68 (0.45, 1.02)	0.66 (0.38, 1.14)	0.76 (0.40, 1.46)	0.99 (0.43, 2.30)
*HEPA* vs. *inactive*	**0.43 (0.27, 0.49)**	**0.53 (0.29, 0.57)**	0.61 (0.29, 1.27)	0.44 (0.17, 1.11)
*Resistance* training vs. inactive	1.04 (0.22, 1.29)	3.82 (0.59, 24.49)	6.22 (0.79, 49.08)	2.05 (0.21, 22.07)
*Aerobic and resistance* training vs. inactive	**0.29 (0.13, 0.64)**	**0.27 (0.08, 0.86)**	0.47 (0.10, 2.01)	0.85 (0.13, 5.41)
*Models’ statistics*				
*LR, p-value*	1432, <0.001	661, <0.001	536, <0.001	388, <0.001
*AIC*	1184.6	699.1	539.1	376.7
*R-square*	0.55	0.49	0.52	0.54
*HEPA, health enchancing physical activity				

Bold font estimates denote significant association at *p* < 0.05. *Model 1* adjusted for age, sex, and MET-minute/week; *Model 2* adjusted for factors included in Model 1 and education, pack-years of smoking, and MedDietScore; *Model 3* adjusted for factors included in Model 2 and BMI, triglycerides, total serum cholesterol and hs-CRP levels; *Model 4* adjusted for factors included in Model 3 and history and management (treated or untreated) of hypertension, diabetes, and hypercholesterolemia. HEPA, health enhancing physical activity; LR, log-likelihood; AIC, Akaike Information Criterion.

## Data Availability

Data described in the manuscript, code book, and analytic code will be made available upon request to the corresponding author.
